# Changing Relationships through Interactions: Preliminary Accounts of Parent–Child Interactions after Undertaking Individual Parent Training

**DOI:** 10.1007/s10560-018-0547-3

**Published:** 2018-05-07

**Authors:** Sarah L. Parry, Jane Simpson, Ste Weatherhead

**Affiliations:** 1 Department of Psychology, Manchester Metropolitan University, Brooks Building, Manchester, M15 6GX UK; 2 DClinPsy, Lancaster University, Lancaster, UK; 3 University of Liverpool, Liverpool, UK

**Keywords:** Communication, Interaction, Attachment, Parent-training, Behaviour

## Abstract

Parent and child interaction training has been increasingly investigated over recent years. However, the mechanisms of change within individual training programmes are not well understood. To explore the factors that can facilitate or inhibit meaningful changes in interactions and ultimately relationships, the current study employed semi-structured interviews to obtain first person accounts from parents who had undertaken an individualised parent-training programme. Three participants provided accounts of the training programme and their perceived impact upon interactions with their children were analysed using inductive thematic analysis. The analysis resulted in three themes, which illustrate how participants adjusted their interactional style with their child to varying degrees through enhanced personal awareness, increased understanding of their child’s emotional and interactional needs, and accepting the reciprocity of interactional accountability. Changes in interactional style enabled participants to alter their perceptions of their own behaviours, their child’s behaviours, and how they influenced one another through interactions. Recommendations for future research and therapeutic practice are discussed in the context of the findings and the existing evidence base.

## Parent Training Programmes

Parent training programmes (PTPs) can be defined as programmes through which “parents actively acquire parenting skills through mechanisms such as homework, modelling, or practicing skills” (United States Department of Health and Human Services, 2009, p. 2). Internationally, the majority of parents/caregivers who request PTPs, or who are advised to undertake parent training, do so because they report struggling to cope with aspects of their child’s behaviour (Egger & Angold, 2006). Group-based PTPs are the predominant intervention for parents and young people (aged 3–14 years) experiencing behavioural difficulties in the United Kingdom (UK, National Institute for Health and Clinical Excellence; NICE, 2017). Such programmes aim to support parents/caregivers in the parent–child relationship through psychoeducation, guided communication and behaviour modelling (Scott & Dadds, 2009).

The majority of PTPs are designed on an assumption that improved social functioning can be achieved through developing an empathic and respectful relationship between the parent/caregiver and child (Pincus, Eyberg, & Choate, 2005) and results are predominantly encouraging (Bjørseth & Wichstrøm, 2016; Engur 2016). Based upon attachment theory (Bowlby, 1958) and social learning theory (Golding 2000), warmth and trust are fostered through the parent/caregiver’s effective modelling, observational skills and praise, or differential attention towards the child’s range of behaviours. Furthermore, consistent boundaries are created through clear age-appropriate directions from the parent/caregiver to the child (Scott & Dadds, 2009). The objective is to reduce behaviours viewed as challenging and address underlying attachment difficulties, which are often illustrated through interactional styles between parent and child.

## Developing Relationships

Attachment theory describes the “lasting psychological connectedness between human beings” (Bowlby 1958, p. 194) and provides a framework for understanding how the development of a child is influenced by their parent, which shapes the parent–child bond. The bond a child forms with their parent can strongly impact upon future relationships (Hooper, 2007) and negatively affect psychosocial functioning if disrupted (Madigan, Moran, Schuengel, Pederson, & Otten, 2007). Attachment styles can alter to some extent over time and “a person’s attachment orientation at any one time is not simply a state or a trait. Instead, it is a combination of influences from contextual factors and enduring ones” (Fraley, Vicary, Brumbaugh, & Roisman, 2011, p. 989). However, research into long-term social networks and emotional health suggests that early relationships affect our relationships with ourselves and others in later life (Charles & Carstensen, 2010), which illustrates the importance of nurturing early relationships and supporting parents to enhance early bonds with their children.

A child will adjust their behaviour depending upon how their main carer interacts with them and young children are thought to “view their collaborative partners [carers] … as intentional, cooperative agents with whom they must coordinate intentional states” (Warneken, Gräfenhain, & Tomasello, 2012, p. 54). Therefore, within the context of attachment theory, a child’s behaviours are representative of their emotions towards their caregiver and designed to elicit a particular response. Attachment based behaviours are selectively designed for the caregiver to express the child’s needs (Schuengel, De Schipper, Sterkenburg, & Kef, 2013). Depending upon how the child’s behaviours are responded to by their caregivers, the child learns how to elicit certain responses from their caregiver. Difficulties within this process are common and parents often report minor difficulties during some early developmental phases (Keenan & Wakschlag, 2000). However, when patterns of learned behaviour become distressing, families may seek help from family intervention services. If group interventions are unsuccessful, families may be referred to more intensive individualised PTPs.

## Individualised Parent Training and Interactional Styles

The current study explores one particular approach to individual parent training, the Parent–Child Game (PCG; Jenner 2008), which Sharry describes as “a systemic individual intervention to reduce behavioural problems in young children” (2004, p. 127). The PCG is based upon an assumption that by establishing a warm and respectful relationship between the parent and child, improved family functioning will follow (Pincus et al., 2005). The warmth and trust in the relationship is nurtured through the parent’s effective observational skills, praise and acceptance, whilst appropriate and consistent boundaries are created through clear directions from the parent to the child. Interactions form the platform for PTPs, such as the PCG, because parent–child communication styles reflect and influence underlying attachment styles (McManus & Poehlmann, 2012; McNeil & Hembree-Kigin, 2010).

A parent’s interactional style can be a significant factor in the development and maintenance of a child’s behaviour and as such is a key element of the PCG process (Nicholson, Fox, & Johnson, 2005). The review of Heinrichs, Cronrath, Degen, and Snyder (2012) highlights the relational aspects of a child’s functioning, the bi-directional nature of conflict between parent and child, and the importance of considering relationships when delivering family focussed interventions. Therefore, greater understanding of the interactional changes that occur through the PCG may illustrate how overall family functioning, attribution of perceived difficulties, and perceptions of interactions alter during the intervention (Naughton & Heath, 2001).

The role of parent–child interactions in child development has also been considered from a neurodevelopmental perspective. Meyer, Wood, and Stanley persuasively suggest that due to the brain’s focus upon development in the first few years of life, and the development of the prefrontal cortex that helps young children understand their emotional experiences, “learning is dependent upon interactions with the environment, including relational interactions.” (2013, p. 165). The emotional attunement between caregiver and child is an important means through which emotional regulation is learned. This interpersonal process that runs interlinked with the developing limbic system in the early years, combined with the interconnected experiences of the child and caregiver, is closely related to attachment theory. Therefore, Meyer, Wood, and Stanley state *nature is nurture* when early brain development is considered in the context of attachment theory and systems theory as they conclude that later life socioemotional functioning is closely linked to the “social and emotional cues from oneself and others” in the early years (2013, p. 164). Specifically in terms of the developing limbic system, the brain’s emotional centre, alongside early relationships, the interplay between neurological and emotional development is clear: “[i]nterpersonal processes (nurture) are essential for physical processes (nature) to occur” (p. 165).

PTPs have been extensively researched in terms of their efficiency for reducing behaviour perceived as challenging (e.g. Martinez & Eddy, 2005; Skotarczak & Lee, 2015) and PTPs that focus on interactional styles can lead to substantial relational change (Chaffin, Funderburk, Bard, Valle, & Gurwitch, 2011; McNeil & Hembree-Kigin, 2010). However, the processes leading to change are poorly understood. In a previous study of two parents who undertook a group PTP, it was suggested that interactional changes could lay “the necessary foundation upon which to bring about behavioural change” (Couch & Evans, 2012, p. 410), although the mechanisms of change and the positioning of PTPs for families engaging with services have only recently been explored (see Cottam & Espie, 2014; Akin & Gomi, 2016). To address the lack of current knowledge around the small changes that occur throughout PTPs, specifically individualised programmes, this study explored whether participants of an individualised PTP (the PCG) reported changes in their interactions with their child and whether interactional changes altered perceptions, tolerance and acceptance towards the child. Consequently, the current study sought to explore mechanisms of change in terms of *how* and *why* interactional change occurs after undertaking the individually delivered PCG.

## Method

### Participant Recruitment

Three White British female participants took part in semi-structured interviews, which ranged from 44 to 108 min. Participants were the main carers for the children with whom they attended the PCG, two mothers and one grandmother, and they were invited to choose their own pseudonyms to protect their identity. The children of the participants were aged between 5 and 9 years old when they accessed the service. The inclusion criteria for the caregivers who took part was that they needed to have attended a minimum of 6 PCG sessions, out of a possible 12. All PCG interventions follow a strict protocol that outlines the preliminary assessments, information provided to parents, content of each session, and how to end the intervention. The homework element of the PCG also involves key tasks including play, star charts or other reward systems, praise and command records, which are reviewed at the beginning of each session. Therefore, by week 6 of the PCG, it was predicted that the fundamental principles of the intervention would have been explained, observed and practised with the participants and clinical psychologists who delivered the programme across locations. Research also indicates that short and abbreviated versions of parent–child training are beneficial for families of young children with challenging behaviour (Nixon, Sweeney, Erickson, & Touyz, 2003). Participants were required to have ended their participation with the PCG no more than 2 years previous to the beginning of this study as research indicates most families are still experiencing the benefits of interventions similar to the PCG at a 2 year follow up (Eyberg et al., 2001). Two participants had attended 12 sessions and one parent had attended 7. Participants were notified about the study through their intervention provider and then contacted the first author directly for further information and to arrange to meet for a research interview.

One possible reason for the limited empirical qualitative research around individualised parent training programmes, such as the PCG, is perhaps participant recruitment. The current study involved two large health trusts and recruited for 14 months. However, only three participants volunteered to participate. This may be due to the barrier of perceived social stigma for parents (Koerting et al., 2013) around accessing individualised parent training, often when other support services and group training have not met their needs, or perhaps a sense of disempowerment that some have cited as potentially inherent to such programmes (Cottam & Espie, 2014). Greater awareness of the impact of stigma on both help-seeking behaviours and participation in research have led to recent calls to consider the influence of self-stigma for parents in care models (Dempster, Davis, Jones, Keating, & Wildman, 2015) and culturally sensitive research (George, Duran, & Norris, 2014) to enhance participation. Consequently, in preparation for data collection, discussions with a parent advisory group were held in order to discuss participant recruitment strategies and to develop the flexible interview schedule.

### Data Collection

The study received ethical approval from the relevant local health authorities and, in accordance with best practice guidelines and research into conducting face-to-face qualitative research with participants (Creswell, 2003; Silverman, 2000), appropriate measures were taken to inform and protect those who took part. All participants were reassured that the provider of their PCG service would not be notified of their involvement or feedback about the intervention. Individual semi-structured interviews were audio-recorded and all documents were stored in accordance with the approved procedure. A flexible interview schedule was developed with seven main questions and associated prompts to encourage reflexive conversation. Questions related to the reasons the participants accessed the PCG, their initial impressions, their interpretation of the approach, their perception of the influence of the PCG for themselves and their child, as well as components of the programme they thought had initiated change. The accounts were transcribed verbatim before the analysis commenced. Interviews were conducted so that the interviews were flexible enough to allow “interviewer and interviewee [to] ‘feed off’ each other as they co-construct data” Gubrium and Koro-Ljungberg (2005, p. 711). This approach accommodates the diverse experiences of the participants in their particular familial milieu.

### Data Analysis

Despite the small number of participants, a thematic analysis was most appropriate due to the varied experiences and systemic circumstances of the participants. Recent considerations around the number of participants required for a thorough thematic analysis, particularly in health and social care, have indicated that smaller numbers of participants are not necessarily a methodological weakness due to the depth of insight and individuality that can be maintained within focused data sets (Fugard & Potts, 2015). Consequently, data saturation and theoretical generalisation were not aims of this study. Rather, the analysis and discussion aim to offer a unique perspective, based on personal accounts of parents and the Information Power model of analysis (see Malterud, Siersma, & Guassora, 2016), to act as a novel platform on which to develop further research to inform service delivery.

An inductive thematic analysis was used due to the exploratory nature of the research question and the flexible utility of the Braun and Clarke (2006) approach with a diverse data set. The analytic approach aided the critical realist conceptualisation and development of emergent themes with the parents’ data (Roth-Yousey, Chu, & Reicks, 2012), accounting for their first-hand interpretations and reinterpretations of the first author, discussed and explored through supervision with the second and third authors. Repeated listening to the audio recordings whilst transcribing provided at least a limited means by which to reduce researcher interpretation and increased the “linguistic meaning within [the] textual material” (Madill, Jordan, & Shirley, 2000, p. 1). In order to develop an inductive set of themes, segments of the data were grouped together according to their topic, to accommodate the variety of participants’ experiences. The 648 codes were classified and cautiously named, forming 26 topic groups describing the emerging themes (Strauss & Corbin, 1990; Tesch, 1990), until an inductive set of four themes grew, two of which were then merged. In summary, the findings of the study provide a synthesised interpretation of participants’ personal experiences of using PCG techniques and the mechanisms that brought about interactional change.

## Findings and Discussion

**Fig. 1:**
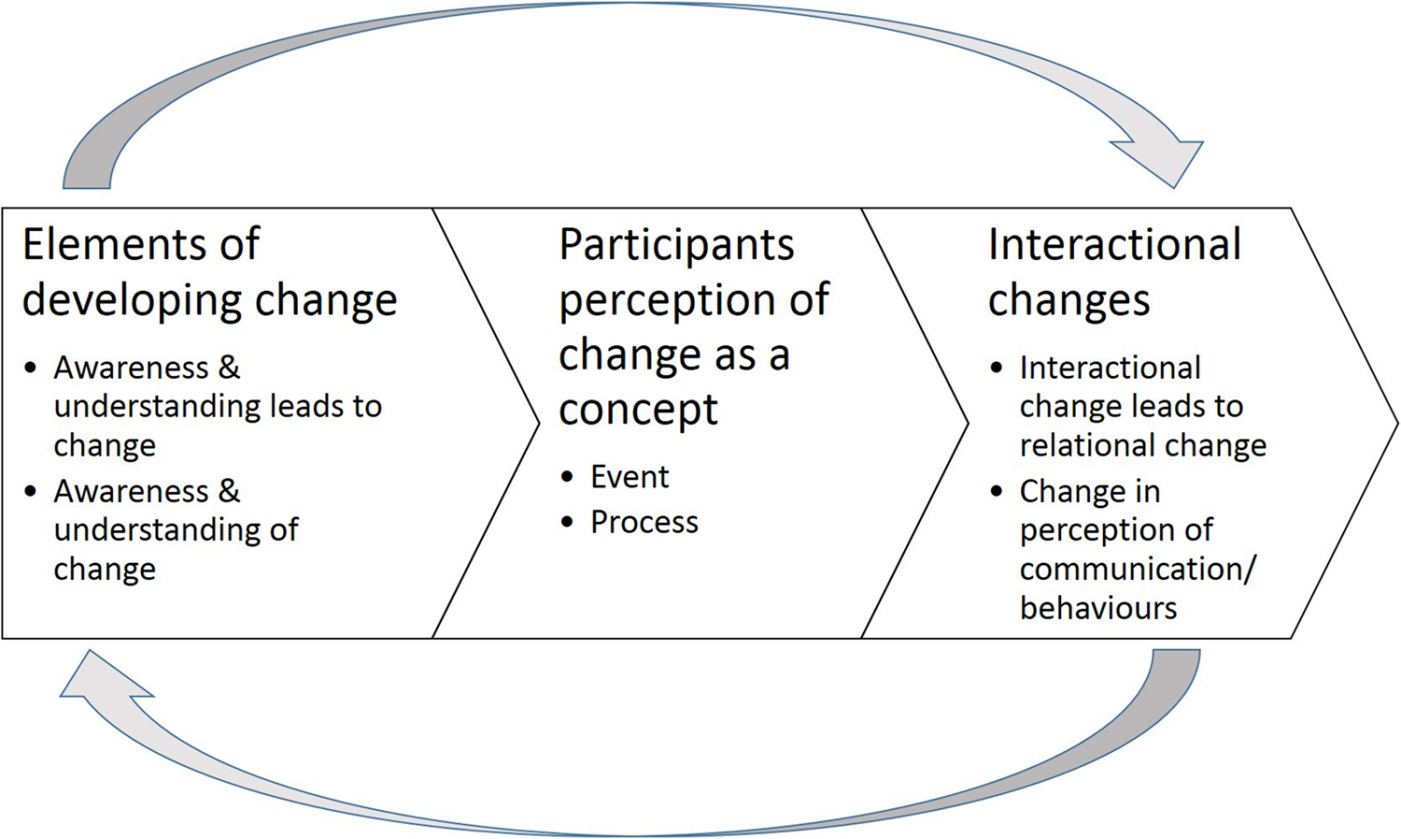
The process of change within parent–child interactions following the PCG across the analysis

The inductive qualitative analysis of the participants’ interviews built three overarching themes. Although the themes are distinct from one another and discuss unique components of the process of interactional and relational change, the overall process of change is illustrated in Fig. 1.

### Theme 1: Developing Awareness and Confidence—“I Guess It Just Makes You Look at Yourself” (Anne)

Developing personal awareness around interactions appeared to facilitate understanding for participants regarding the role they played in interactions with their child. For example, Liz became aware of the connection between how she felt in the moment and how she spoke to her son, Jake. Liz explained that when she was aware of how her emotions influenced her communicative style and how her approach affected Jake’s emotions, she commented: “the way you deal with things, your child’s taking all that”. Similarly, Anne described how the PCG “really does make you aware of your actions”, as she reflected that the PCG process had enhanced her awareness of her coping strategies in response to other stressors she was managing in day-to-day life.

Participants reported their increased awareness of their emotions and resulting communicative behaviours facilitated a more detached mindful watching of their interaction style. Liz discussed that “it just made me aware of what I was actually doing with my child, you know, umm bombarding him all the time really, instead of just letting him lead”. Consequently, Liz would wait to be invited to interact with Jake: “It [the PCG] made me aware of constant interruptions, which I was doing”. Singh et al., discusses the impact of mindfulness for PTPs, suggesting that mindfulness training encourages “personal transformation” (2010, p. 157) rather than only the learning of strategies, which has a positive influence on parenting satisfaction and reduces conflict. Although mindfulness training is not an explicit feature of the PCG, it appears the reflections on progress and self-monitoring component of the latter half of the programme encouraged reflexivity and awareness of emotions and actions to occur. In monitoring her own actions, Margaret found that praise was more effective for Lucy than ignoring less desirable behaviour: “it’s just knowing the right ways to praise her and encourage her”, whereas Liz found less could be more: “step back, don’t ramble on, firm commands, following it through”. Accordingly, participants were able to establish interactional change through altering their communications, conveying a contained or positive message, rather than an emotion, by: “changing it… the way you say things” (Liz).

Therefore, an important feature of adapting communication was to convey a message, rather than intense emotion. All participants discussed how they had learnt to be increasingly responsive and less reactive. For example, Margaret explained how “you can’t praise someone who’s pinching you, so you get the next available moment”. Liz recognised her slower approach enabled her to feel more in control, so that she could be more considered and responsive when Jake invited her to play, commenting: “getting your self-confidence back, and once you’re in control, then everything falls into place”. Although Anne also found some strategies helpful, she did not describe feelings of empowerment and discussed throughout her interview that she “was not convinced” by the PCG approach: “Just praising the positive really and trying your best to ignore it, but it is difficult.” Tentatively, it could be interpreted that the strategies alone, without a sense of empowerment, led to less overall positive change. Witnessing positive change and gaining confidence, perceived control and calmness seemed inextricably connected with empowerment: “I feel more in control, that was a big thing… even now I get so I can’t cope but he can be very challenging… It’s given me more confidence” (Liz).

An aim of the PCG is to help participants develop new skills and strategies, which they can learn in clinic and apply at home. All participants reported finding ‘how to’ strategies, facilitating communicative adjustments. For example, Margaret explained how processes within the PCG assisted the implementation of interactional adjustments: “It’s the structure really… that you can be guided through the sorts of praise, and when to ignore and when it needs further intervention”. Similarly, Liz described understanding why her interactions with Jake needed to change: “it’s [the PCG] made me realise that it’s too much for your brain to go with and let them lead and then you can join in if they want you to join in” (Liz). Consequently, through developing an “increase in feelings of empathy, understanding and acceptance” (Barlow & Stewart-Brown, 2001, p. 127) through play, Liz and Margaret created new patterns for communication, bringing about relational change. In summary, responding to the child rather than reacting to a behaviour, and enhanced acceptance stemming from increased awareness of how their own emotions influenced their children, participants nurtured warmth within interactions, as Margaret explained: “it just needs bringing out”.

### Theme 2: Making Changes—“It’s All Psychological” (Liz)

Developing new ways of coping with challenges was an important feature within the accounts, as participants described the difficulties they faced before the PCG intervention: “before I just couldn’t cope with it all” (Liz). Liz explained how her new skills enhanced her ability to cope: “You can make it easier for yourself but you just need to know how”. Additionally, Margaret noticed that when she communicated the *why* in interactions, Lucy developed her own emotional understanding of a situation, thus reducing the likelihood of conflict: “Whereas before, just a general ‘good girl’, it’s not enough, you have to let them know *why*”. Thus, explaining behaviours scaffolded emotional understanding and reduced overall conflict.

Participants also discussed how difficulties they had faced in their child’s early years affected their present relationships: “at first we didn’t have a bond” (Liz). However, changes participants were trying to make could be difficult to maintain when they felt stressed or when other factors in the home environment acted as barriers: “If you put it into the real world it just won’t work … Liam sitting there having one to one, that’s not a real family, that’s not how it operates” (Anne). Margaret, in particular, evidenced the impact of stress, discussing the impact of her own health and medical appointments upon Lucy: “I weren’t prepared for her sliding backwards…” Research suggests that when one is increasingly mindful and aware throughout emotive situations, we are less likely to repeat the intergenerational transmission of relational schemas (Bögels, Lehtonen, & Restifo, 2010; Siegel & Hartzell, 2004), although as Anne explained, this could be very difficult: “when you look at your parents, you do as a parent what they did, what you see as normal”. The current study indicates that personal awareness and empowerment could support stress resilience and therefore further interactional change, although environmental barriers at home could prevent progress.

Changes in participants’ perspectives towards their child’s behaviour influenced how participants viewed the exchange of behaviours in interactions. For instance, Liz was able to alter her perspective on Jake’s behaviour, contextualising their experiences as a family and stressors that affected Jake at home and at school, which she then endeavoured to explain to other family members “just to make them understand”, so they could support Jake as a family. Liz also described how her own emotional difficulties influenced their relationship: “So the way you deal with your child is about your life stresses and you need to think about that, you know the way you’re feeling, taking it out on your child.” One of the ways in which Liz found she could take a step back and maintain her mindful awareness was to “let them [children] lead” (Liz). When participants’ perspectives of their own behaviour and their child’s behaviour were formulated in a developmental psychosocial context, they were able to find a more empathic and accepting position, which influenced their interactional style. It has been suggested that being an empathic parent is the most important factor to influence the psychological wellbeing of a child (Barlow & Stewart-Brown, 2001; Bavolek, 2014). For participants of this study, interactional and relational changes enhanced the overall functioning of the family unit, which could facilitate greater insight and empathic perspectives on behaviour to sustain positive change.

However, transitioning the PCG strategies from the clinic to home could be difficult, especially for Anne who was caring for a number of young children. She explained how her son “thrived on one-to-one” but that it could be difficult to provide protected one-to-one time at home with little support: “there are so many of us and they’re all fighting for attention it’s difficult to give them all the individual attention they all crave”. Similarly, Liz stated, “If we could have gone on it as a family that would have been helpful…” Margaret saw greater systemic change, explaining: “she’s [Lucy] getting on better with her siblings now. She’s now sharing a bit more”. Margaret and Liz described developing a comprehensive understanding of the PCG strategies and attended all of their later sessions, which may have provided time to reflect on their progress, consolidate the strategies in clinic, gain confidence, and facilitate further change at home. PTPs that involve reflective elements, such as the PCG, as well as systemic support towards the end of the intervention have been found to be particularly effective (Dunlap et al., 2006; Shonkoff & Phillips, 2000), a finding replicated here. Although all participants reported positive degrees of change, support at home was recognised as an important component from which they would have benefited; as Anne explained: “could have had more family involvement, not just me and Liam, maybe some sessions with the family unit”.

### Theme 3: Experiencing Change—“There’s Been Quite a Large Shift” (Anne)

Although the concept of change was discussed by all participants, the nature of change varied among them. Margaret recognised that: “it’s a limited working… every area is say 60–70% better”. Despite recent setbacks, Margaret explained: “Well, all the advice was brilliant and following it is brilliant… most of the time they [the strategies] do work”. For Anne, difficulties at home presented obstacles to implementing strategies, possibly compounded by tensions in Anne’s relationship with her PCG practitioner. Difficulties in the therapeutic alliance in PTPs can lead to negative outcomes overall (Greef, Pijnenburg, van Hattum, McLeod, & Scholte, 2017) and, among other psychosocial and economic barriers (e.g. Akin & Gomi, 2016), the therapeutic relationship has been cited as a potential cause for early discontinuation with services (Barth et al., 2005; Harwood & Eyberg, 2004), as in Anne’s case. The common disparity between the environmental conditions in clinic and participants’ home environments has prompted a call for PTPs to “fit with the reality of family life today” (Department for Children, Schools and Families, 2010, p. 4).

Although Liz reported ongoing difficulties in her relationship with Jake, she discussed feeling empowered and coping. This change in her confidence may suggest why she felt as though the PCG had worked: “so what worked for me was, I would just sit back and say I’ll just be here and if you want me to join in just say and that worked for us you know”. These degrees of change support the use of training around attachment and social learning techniques through PTPs (Brestan, Eyberg, Algina, Johnson, & Boggs, 2003), although the participants’ family context and relationship with the PCG practitioners also appeared key to maintaining change.

Finally, endings were experienced differently by all participants. Towards the end of her interview, Anne concluded her reasons for not completing the programme, reflecting: “It’s hard cos it’s not natural. I understood it and why we had to do it, praise the positive and ignore the negative. I completely understand that but it’s just not natural…” Throughout Anne’s interview she explained how the intergenerational transmission of parenting strategies, the demands upon her time caring for a number of young children, a difficult working relationship with her PCG practitioner, and lack of external support all contributed to early termination of the sessions and a sense that more could have been done. However, she also commented on the positive elements of the programme, which had facilitated one-to-one time with her son in the clinic setting “It were nice to see him smile, see him matter.”

Conversely, Liz reported a very positive relationship with her service provider. She described how she valued the changes her practitioner witnessed, which seemed to give Liz a sense of validation: “she [therapist] could see how more confident and in control I am, and I think that for me is a lot to do with it”. Margaret had a more complicated ending due to her deteriorating physical health, although remained optimistic she could practise the techniques in the long-term, advising: “Just take it on board what they say, because you know, it does work”. In summary, participants who identified as coping and as having support from older children or relatives used the techniques for longer. Therefore, exploring wider family functioning during individual PTPs may help parents/caregivers and clinicians develop a transition plan supporting relevant, achievable strategies. Such an approach could have supported Anne further, even though the clinic sessions ended prematurely. Participants also placed more importance on family involvement towards the end of their training, supporting the transition from clinic to home. Consolidating PCG strategies towards the end of training was a positive factor in clinic to home transitions, with space for reflexive endings supporting the awareness of change and progress, which enhanced participants’ confidence and sense of empowerment. Dumas (2005) suggested that when interactions become less automatized and more mindful, change can more readily occur. However, the findings reported in this study suggest that even when interactions are mindful, if the wider system around the two people in the interaction remains automatized, as Anne reported, change is more difficult to instigate.

## Discussion and Practice Recommendations

This study aimed to explore how an individualised PTP could support interactional change and highlights important transformations at an intrapersonal and interactional level within parent–child dyads, and tentatively, within wider family systems. To varying degrees, participants were able to develop alternative interactional patterns with their children through understanding and utilising “mechanisms of change” (Bögels et al., 2010, p. 107). The mechanisms found in this study were focussed attention upon the parent–child interaction in the moment; mindful awareness of one’s own emotions; conveying a message rather than an emotion; using age-appropriate emotionally informed language; making connections between emotions and communication; and scaffolding emotional understanding for the child.

Understanding these interactional subtleties helped participants take a longer-term approach to interactions, prioritising positivity and diminishing negative bias. This approach meant that participants could convey warmth from a position of acceptance, creating a platform for empathy and understanding. These personal accounts add a new depth of insight and detail to existing empirical PTP research. Perhaps of particular importance, it was the participants’ awareness of their own emotions, rather than their children’s behaviours, which helped foster understanding of the reciprocity of interactions and reduced conflict. Additionally, through understanding their own emotions and behaviours, participants became able to scaffold emotional understanding for their children, which appeared to increase acceptance and reduce conflict further. This finding emphasises the importance of PTPs in helping parents understand the role of emotions in interactions and in their child’s development, rather than focussing on only adjusting the behaviour of their child.

In terms of participants’ reflections on the programme, the systemic elements appeared an important aspect for development. Participants’ perceptions of interactional change seemed to be a combination of their experiences of the PCG and what they could reproduce of those learnt experiences at home. The analysis suggests that systemic support is particularly important towards the end of training, rather than solely in the planning stages. Finally, the findings of this study suggest that even when interactions are mindful, if the wider system around the two people in the interaction remains automatized, change is more difficult to instigate and maintain. Consequently, systemic support and interactional change within the wider family system appears to be an imperative next step for exploration for individualised PTPs.

Despite the novelty of this study, there were some limitations. A greater number of participants may have provided greater variation within the data and further examples of influences within individual environments. However, the aforementioned use of the Information Power model of analysis (Malterud, Siersma, & Guassora, 2016), which values the novelty of data alongside the aims of the study and quality of researcher-participant dialogue, goes some way to mediate this limitation. Additionally, Liz and Margaret completed the PCG, while Anne completed seven of the 12 sessions. Consultation with service providers indicated that most of the training elements are presented in the first six sessions, meaning that all participants received the majority of the instructional components of the PCG. This is representative of findings in larger samples, which indicate around one-third of participants end their engagement with PTPs before the final session (e.g. Akin & Gomi, 2016). Finally, this study was not successful in recruiting fathers, which is a generally recognised gap in the evidence base for PTPs (Barlow, Smailagic, Huband, Roloff, & Bennett, 2012) or parents/caregivers from ethnic minority groups (see Butler & Eyberg, 2006). However, this is a unique small-scale study, qualitatively exploring mechanisms of change in parent–child interactions through individual parent training, which has indicated a number of recommendations for practice and further study.
